# Enhanced Charge Extraction of Li-Doped TiO_2_ for Efficient Thermal-Evaporated Sb_2_S_3_ Thin Film Solar Cells

**DOI:** 10.3390/ma11030355

**Published:** 2018-02-28

**Authors:** Chunfeng Lan, Jingting Luo, Huabin Lan, Bo Fan, Huanxin Peng, Jun Zhao, Huibin Sun, Zhuanghao Zheng, Guangxing Liang, Ping Fan

**Affiliations:** 1 Shenzhen Key Laboratory of Advanced Thin Films and Applications, College of Physics and Energy, Shenzhen University, Shenzhen 518060, China; lanchunfeng@gmail.com (C.L.); luojt@szu.edu.cn (J.L.); lanhb420@163.com (H.L.); fanb07@hotmail.com (B.F.); P2385284535@163.com (H.P.); zhaojun@szu.edu.cn (J.Z.); hbsun@szu.edu.cn (H.S.); zhengzh@szu.edu.cn (Z.Z.); 2 Key Laboratory of Optoelectronic Devices and Systems of Ministry of Education and Guangdong Province, College of Optoelectronic Engineering, Shenzhen University, Shenzhen 518060, China; 3 Institute of Thin Film Physics and Applications, College of Physics and Energy, Shenzhen University, Shenzhen 518060, China

**Keywords:** Li-doping, charge extraction, thermal evaporation, Sb_2_S_3_ solar cells, photovoltaic performance

## Abstract

We provided a new method to improve the efficiency of Sb_2_S_3_ thin film solar cells. The TiO_2_ electron transport layers were doped by lithium to improve their charge extraction properties for the thermal-evaporated Sb_2_S_3_ solar cells. The Mott-Schottky curves suggested a change of energy band and faster charge transport in the Li-doped TiO_2_ films. Compared with the undoped TiO_2_, Li-doped mesoporous TiO_2_ dramatically improved the photo-voltaic performance of the thermal-evaporated Sb_2_S_3_ thin film solar cells, with the average power conversion efficiency (*PCE*) increasing from 1.79% to 4.03%, as well as the improved open-voltage (*V_oc_*), short-circuit current (*J_sc_*) and fill factors. The best device based on Li-doped TiO_2_ achieved a power conversion efficiency up to 4.42% as well as a *V_oc_* of 0.645 V, which are the highest values among the reported thermal-evaporated Sb_2_S_3_ solar cells. This study showed that Li-doping on TiO_2_ can effectively enhance the charge extraction properties of electron transport layers, offering a new strategy to improve the efficiency of Sb_2_S_3_-based solar cells.

## 1. Introduction

Investigation of low-cost, abundant, and efficient absorbing materials is one of the most important issues for solar cell applications. From this aspect, chalcogenide compounds, such as Sb_2_S_3_, Sb_2_Se_3_, have attracted considerable attention in recent years [1,2,3,4,5,6]. Compared to Sb_2_Se_3_, Sb_2_S_3_ exhibits some unique merits, such as the tunable band-gap with high absorption coefficient, easy processing, stability and with abundant raw materials [2,3,4,5]. More importantly, the Sb_2_S_3_-based solar cells show excellent photovoltaic performance in weak light illumination conditions, which makes it feasible to achieve efficient power conversion in cloudy days or indoor conditions [7]. Therefore, the research on high-performance Sb_2_S_3_ solar cells is still of high value.

The Sb_2_S_3_ films for solar cells application have been prepared by thermal evaporation and chemical deposition method [3,8,9,10,11,12]. Compared to the chemical deposition, thermal evaporation shows some unique advantages, e.g., compatibility with future fabrication of large-area solar cells [8,12]. However, so far, the thermal-evaporated Sb_2_S_3_ thin film solar cells only showed a highest *PCE* of 3.01%, which is lower than that of chemical deposition method [10,12]. The thermal-evaporated Sb_2_S_3_ solar cells are still encountering the problem of low *PCE*, as well as the low short-circuit current and open-circuit voltage [10,11]. How to improve their photovoltaic performance remains a priority for Sb_2_S_3_ solar cells [7,13,14,15]. It is reported that besides the film quality of absorbing layers, low charge extraction was another important factor affecting the power conversion of devices. In particular, the transport efficiency of photo-excited electrons from the absorbing layer to the electron transport layer was very low on the Sb_2_S_3_/TiO_2_ interface, which impeded the improvement of the power conversion efficiency [16]. To overcome this limitation, some new materials and structures have been introduced into the Sb_2_S_3_ solar cells, such as ZnO and ZnO cored TiO_2_ rods as electron transport layers to enhance the charge extraction process [17,18,19,20]. Unfortunately, so far few of the Sb_2_S_3_ solar cells based on these materials or structures reached a satisfying *PCE*, leaving many challenges for future development [17,18,19,20]. Thus, new strategies are required to raise the photovoltaic performance of Sb_2_S_3_ solar cells. Recently, doping of TiO_2_ layer has been reported as an effective method to improve the charge extraction in perovskite solar cells [21,22,23]. This gives us an inspiration for the improvement of Sb_2_S_3_/TiO_2_-based solar cells. 

In this work, we use the thermal-evaporated Sb_2_S_3_ thin films as absorbing layers and Li-doped TiO_2_ as charge transport layers to fabricate the solar cells, and we find that Li-doping dramatically improves the photovoltaic performance of Sb_2_S_3_ solar cells, with an average *PCE* of 4.03%, as well as a champion *PCE* up to 4.42% and *V_oc_* of 0.645 V, offering an efficient method to raise the photovoltaic performance of thermal-evaporated Sb_2_S_3_ solar cells.

## 2. Experimental

### 2.1. Preparation of Sb_2_S_3_ Thin Film and Li-Doped TiO_2_

Sb_2_S_3_ thin films were thermally evaporated on corning glass substrates or TiO_2_-coated fluorine-doped SnO_2_ glass (2.0 × 2.0 cm^2^, fluorine-doped tin oxide (FTO) glass, Sigma-Aldrich, Saint Louis, MO, USA) under a high vacuum (1.5 × 10^−3^ Pa) using 0.5 g of commercial Sb_2_S_3_ powder (99.999%, Sigma Aldrich). The evaporation was carried out at room temperature. After the evaporation, the as-deposited films were immediately transferred into N_2_ glove box and annealed on hot plate at 275 °C for 10 min. As mentioned in reference [24], the TiO_2_ precursor was spin-coated on the corning glass and FTO glass respectively, and then sintered at 450 °C for 30 min to form a compact layer. TiO_2_ paste (30NR-D, Dyesol, Queanbeyan, Australia) diluted by alcohol (weight ratio of 1:6) was spin-coated on a dense TiO_2_ compact layer, and then sintered at 450 °C for 30 min to form a mesoporous structure. For the Li-doped mesoporous TiO_2_, 0.05, 0.1, 0.2 M bis (trifluoromethane) sulfonimide lithium salt (Li-TFSI) (99.9%, Macklin, Shanghai, China) in acetonitrile solutions were spin coated on the meosporous TiO_2_ layers respectively (with the Li-TFSI salt concentration higher than 0.2 M, the homogeneity of the TiO_2_ film was sharply reduced, thereby the concentration of Li-TFSI salt used was not higher than 0.2 M), and again sintered at 450 °C for 30 min to form Li-doped TiO_2_. After cooling to 150 °C, the substrates were immediately transferred into the thermal evaporator for the deposition of Sb_2_S_3_ thin films.

### 2.2. Device Fabrication

FTO glass was cleaned by isopropanol, acetone, de-ionized water, and alcohol in ultrasonic cleaner, dried and then treated by ultraviolet ozone treatment before use. Li-doped TiO_2_ films were prepared on cleaned FTO glass as described above. In addition, then the Sb_2_S_3_ films were thermally evaporated on them and annealed in an N_2_ atmosphere glove box. 72.3 mg of Spiro-OMeTAD in 1 mL of chloridebenze solution was used as hole transport materials with the addition of 28 µL of 4-tert-butylpyridine and 19 µL of TSFI-Li acetonitrile (520 mg/mL). It was spin coated on the Sb_2_S_3_ films at a speed of 3000 rpm for 30 s. Finally, 50 nm of Au film was deposited as top electrode by thermal evaporation. The devices fabricated on the different Li-doped TiO_2_ were labeled as undoped TiO_2_, 0.05Li + TiO_2_, 0.1Li + TiO_2_ and 0.2Li + TiO_2_.

### 2.3. Characterization

The phase structure of the films was analyzed using powder X-ray diffraction (XRD) (Ultima IV, Rigaku, Tokyo, Japan) with Cu*Kα* radiation (λ = 0.15406 nm) operated at 40 kV and 40 mA. The surface morphology and cross-section of prepared films and devices was analyzed by field-emitted scanning electron microscopy (FE-SEM) (SUPRA 55, Zeiss, Oberkochen, Germany). The composition of the film was mapped by the energy dispersive x-ray microanalysis system (EDX) (Bruker QUANTAX 200, Bruker, Billerica, MA, USA). X-ray photoelectron spectroscopy was measured by using a system (PHI 5000 Versa ProbeⅡ, Ulvac-Phi, Chigasaki, Japan) with a monochromatic Al *Kα* X-ray source (1486.7 eV) at 50 W and 16 kV with a beam spot size of 200 μm. UV-visible spectra measurement was performed by a spectrophotometer (UV-3600Plus, Shimadzu, Japan). Mott-Schottky measurement was carried out using an electrochemical workstation (CHI660E, CH Instruments, Shanghai, China) with the structure of FTO/TiO_2_, at a scan rate of 10 mV∙s^−1^. Current density-voltage (*J-V*) characteristics of the Sb_2_S_3_ solar cells were tested under simulated AM 1.5G conditions (100 mW/cm^2^) with a Keithley 2400 sourcemeter in ambient condition in-house. The voltage was scanned from 0 to 1 V with a scan rate of approximately 0.1 V/s. Devices area illuminated were precisely set by a mask with an area of 0.08 cm^2^. External quantum efficiency (EQE) was measured with the photoelectric conversion test system (SCS100-X150-DSSC, Zolix Instruments, Beijing, China) with a standard silicon solar cell as reference.

## 3. Results and Discussion

**Figure 1:**
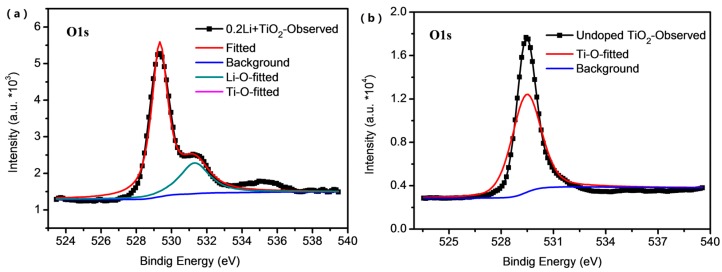
X-ray photoelectron spectroscopy. (**a**) The O1s peaks fitting for 0.2Li + TiO_2_ sample. (**b**) The O1s peak fitting for undoped TiO_2_ sample.

With the Li-TFSI deposition and sintering processes, the introduction of lithium lead to the different surface states of mesoporous TiO_2_ layers, such as the formation of LiO_2_, LiOH or Li_4_Ti_5_O_12_ [25,26], which sharply affects the electron extraction properties of the TiO_2_ layer in the perovskite solar cells. To study the elemental states in the undoped and Li-doped mesoporous TiO_2_ layers, X-ray photoelectron spectroscopy (XPS) was applied to characterize the elemental compositions. Figure 1 shows the fitted XPS patterns of the TiO_2_ and the 0.2 M Li-doped TiO_2_ films. Generally speaking, the XPS intensity of the Li-doped TiO_2_ is apparently weaker than that of the undoped samples, inferring that the surface state of TiO_2_ layers has been changed by the lithium-treatment. In detail, in Figure 1a there is a slight shoulder at the peak near 530 eV in the XPS patterns of the Li-doped TiO_2_ film, where the peak is related to the O1s spectra [22]. The peak deconvolution suggests that this shoulder originated from the interaction of the oxygen and the lithium [25]. For undoped TiO_2_, Figure 1b only shows a peak related to the Ti^4+^. There is no obvious finding on spectra difference in detailed fine scanning on the Ti 2p region (Figure S1). The lithium doping on TiO_2_ can result in the reduction from Ti^4+^ to Ti^3+^, which can passivate the trap state or defects in TiO_2_ films [22,23]. Correspondingly, this passivation can improve the charge transport in the lithium-treated TiO_2_ [26].

**Figure 2:**
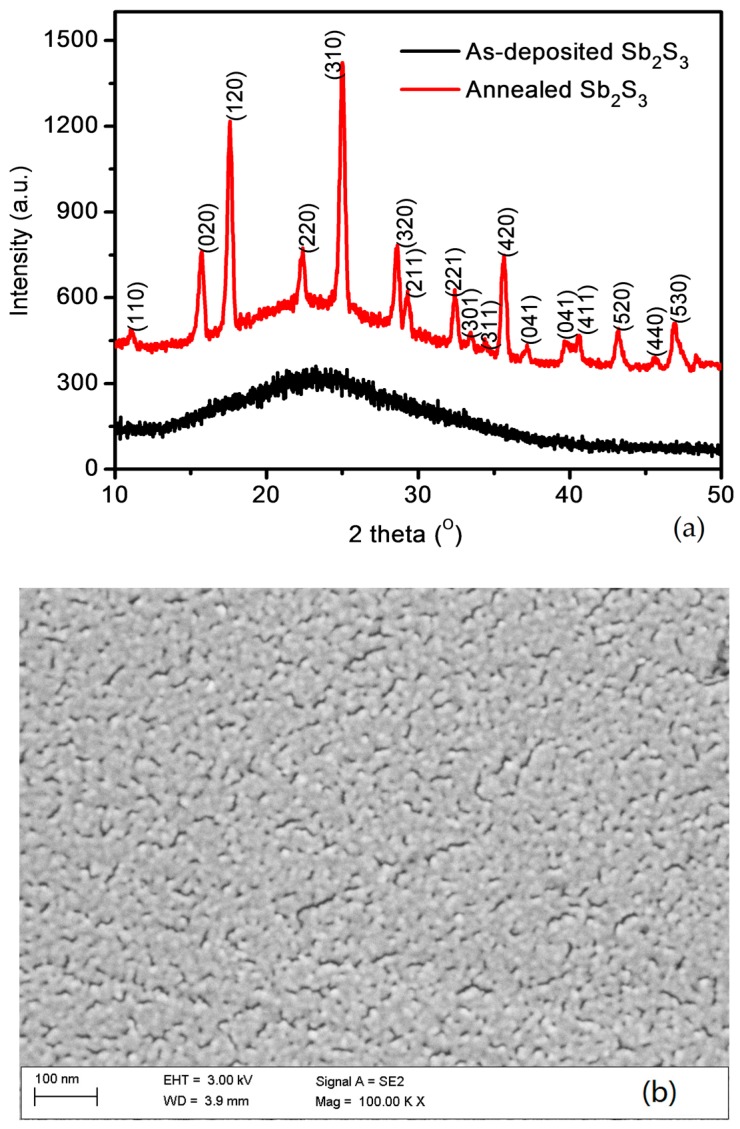
(**a**) XRD patterns for the Sb_2_S_3_ thin films and (**b**) the SEM of the surface morphology of the annealed Sb_2_S_3_ films.

Figure 2a shows the XRD patterns of the as-deposited film and the annealed Sb_2_S_3_ film. It clearly shows that the as-deposited film is in amorphous state. For the sample annealed at 275 °C, the XRD pattern indicates a typical orthorhombic stibnite Sb_2_S_3_ phase (PDF#42-1393). The full width at half maxima (FWHM) for the peaks (310) is 0.360. Accordingly, the crystalline size calculated from the Debye-Scherrer formula for the peaks of (020), (110), (310) and (420) are 22.03, 24.46, 22.36 and 24.62 nm, respectively [27]. Figure 2b shows the SEM images of the surface morphology of the annealed Sb_2_S_3_ film. The uniform nano-crystalline of Sb_2_S_3_ films was formed after the thermal annealing. The thermal annealing process is accompanied by the obvious changes of crystalline and optical properties [4,8]. The Sb_2_S_3_ films annealed at 275 °C showed relatively small nanograins and homogenous surface morphology, which might reduce the leakage currents of devices. Figure S2 displays the EDX results. The atomic ratio of Sb:S is approaching 1:1.5, a bit larger than that of the ideal defect-free Sb_2_S_3_. The change of atomic ratio might come from the evaporation and the annealing process. It was reported that the thermal annealing of Sb_2_S_3_ films is accompanying with the sulfur diffusion, and with higher temperature and longer annealing time, the sulfur is more likely to diffuse into the environment with sulfur defects left in the films [8]. Moreover, because nanocrystalline Sb_2_S_3_ is enough for an efficient Sb_2_S_3_ solar cells [4], the annealing temperature of 275 °C was used for the crystallization in our experiment. From the EDX result the annealed Sb_2_S_3−x_ film shows some sulfur-vacancies, suggesting the formation of N-type Sb_2_S_3_ absorber.

**Figure 3:**
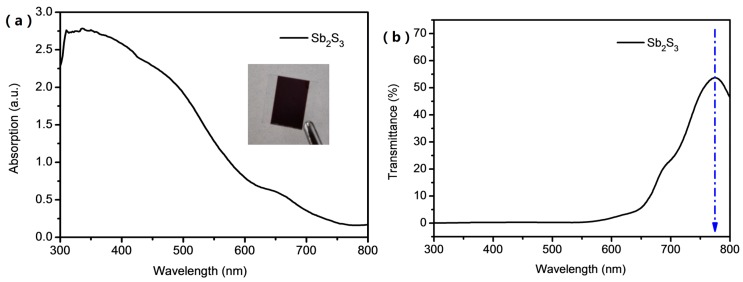
(**a**) UV-visible light absorption of the Sb_2_S_3_ thin films (the inset picture is the sample of the evaporated Sb_2_S_3_ thin film) and (**b**) transmittance spectrum of the Sb_2_S_3_ thin film.

Figure 3a shows the UV-visible light absorption of the annealed Sb_2_S_3_ film. The as-deposited amorphous state Sb_2_S_3_ film shows the color of yellow brown (Figure S3). After thermal annealing, the crystallized films become dark brown with the change of crystal structure (as shown in the inset picture). As shown in Figure 3a the absorption of the annealed Sb_2_S_3_ film covers the visible light region. The optical bandgap of the Sb_2_S_3_ thin film is estimated from transmittance spectrum in Figure 3b [28], where the threshold of the crystallized Sb_2_S_3_ films is 775 nm, indicating a bandgap *E_g_* of 1.6 eV. However, even the threshold starts near 775 nm, the major absorption in the visible light region is mainly located in the range of 300 to 600 nm. Additionally, a small absorption tail is observed near infrared region. According to the XRD and SEM results, it must be the Urbach energy tail attributed to some amorphous state in the Sb_2_S_3_ films [4,29,30].

**Figure 4:**
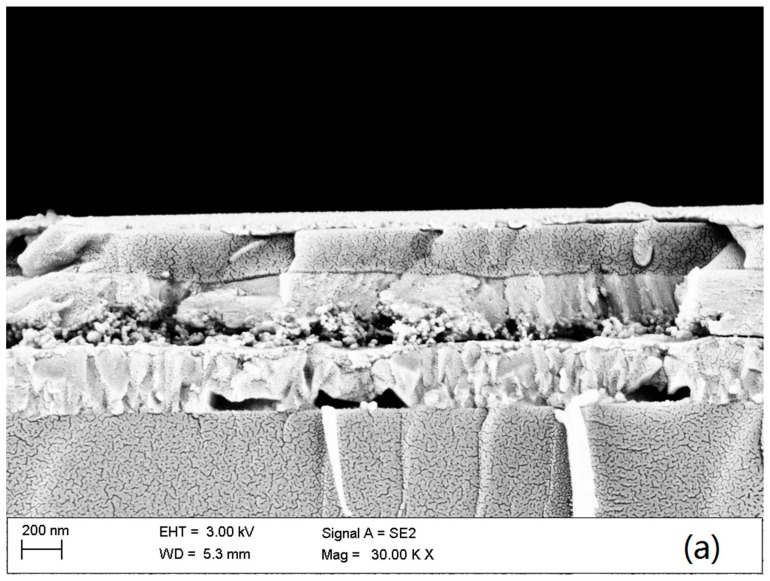

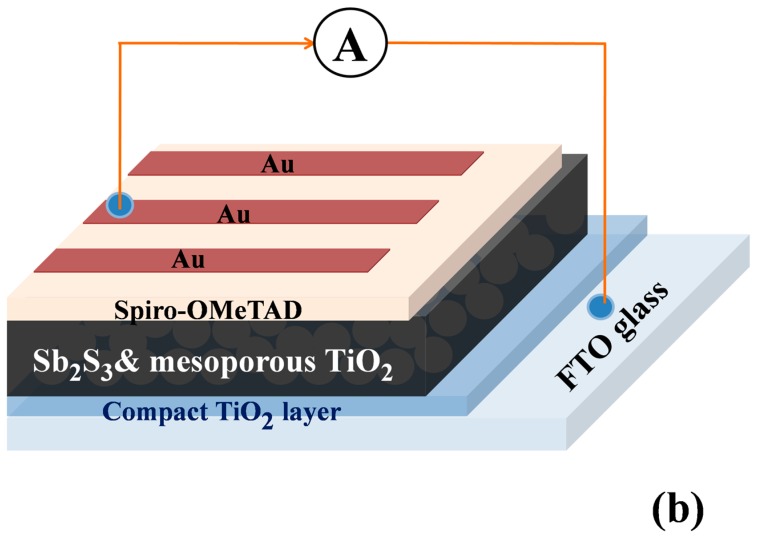
(**a**) Cross-section SEM of the Sb_2_S_3_ solar cells and (**b**) the configuration of the device.

**Figure 5:**
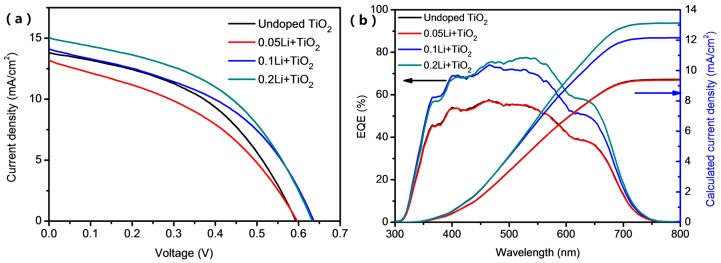
(**a**) The champion *J-V* curves of the devices based on different Li-doped TiO_2_ and (**b**) EQE of the Sb_2_S_3_ solar cells.

To check the lithium doping TiO_2_ effect on the photovoltaic performance of the Sb_2_S_3_ solar cells, the thermal evaporated Sb_2_S_3_ solar cells with the Li-doped TiO_2_ electron transport layers were fabricated. Figure 4a shows the cross-section SEM image of the devices, and Figure 4b shows the configuration of the device in an architecture of FTO/compact TiO_2_/mesoporous TiO_2_/Sb_2_S_3_/HTM/Au. The thickness of mesoporous TiO_2_/Sb_2_S_3_ is approximately 310 nm, where the mesoporous TiO_2_ layer is 100 nm thick. In addition, the Spiro-OMeTAD layer is 200 nm thick. The cross-section SEM of the devices indicates the dense homogenous structures are formed in our experiment. In addition, then the photocurrent density–voltage characteristic was conducted under standard AM 1.5G one Sun illumination. Figure 5a shows the current density-voltage curves of the champion devices in each group. In the thermal-evaporated Sb_2_S_3_ solar cells with the undoped TiO_2_, we reached a champion *PCE* of 3.74%, higher than that of the thermal-evaporated Sb_2_S_3_ solar cells previously reported (1.27% and 3.01%) [10,12]. It could be explained that the sulfur vacancies in the absorbing film resulted in higher concentration of electrons than that in defect-less or N-type Sb_2_S_3_ films, which benefits the overcoming of the heavy effective electron mass of the intrinsic Sb_2_S_3_ films. Additionally, from photovoltaic parameters in Table 1 and Figure S4 we clearly found that the photovoltaic performance of the devices was apparently improved with the increased doping of lithium on the mesoporous TiO_2_ layers. The average *V_oc_* of the Sb_2_S_3_ solar cells increased from 0.591 to 0.629 V with the increase lithium doping on meso-TiO_2_, inferring less energy loss of the photo-excited electrons. At the same time, the average *PCE* increases from 1.79% to 4.04% with the increasing shunt resistances (*R_sh_*) and the reduced series resistances (*R_s_*). The *EQE* of Sb_2_S_3_ solar cells is shown in Figure 5b, and the calculated current density is accompanying with the tendency listed in Table 1. The lithium doping on the mesoporous TiO_2_ can result in the enhanced electron extraction of TiO_2_ [22,23]. Correspondingly, the faster electron extraction can happen from Sb_2_S_3_ to TiO_2_ layer, which reduces the recombination of electron-hole pairs in the absorbing layers. The statistics in Table S1 and Figure S3 further demonstrate that the devices based on the Li-doped TiO_2_ are more repeatable, and their photovoltaic performance varies less than that based on the undoped TiO_2_. The TiO_2_ layers may become more conductive after Li-doping [22,23], thus the devices can achieve more effective charge extraction and the increase of the average short current density from 10.4 mA/cm^2^ to 14.3 mA/cm^2^, as well as the fill factors obviously improved from 0.28 to approximately 0.45. The Sb_2_S_3_ solar devices with undoped TiO_2_ only showed an average *PCE* of 1.79%, while the devices with the highest lithium doping exhibited the best power conversion performance with a champion *PCE* of 4.42%, with the average *PCE* of 4.03%. The Sb_2_S_3_ solar cells based on 0.2Li + TiO_2_ achieved a *V_oc_* of 0.645 V (as shown in Figure S5), much higher than that with the undoped TiO_2_.

**Figure 6:**
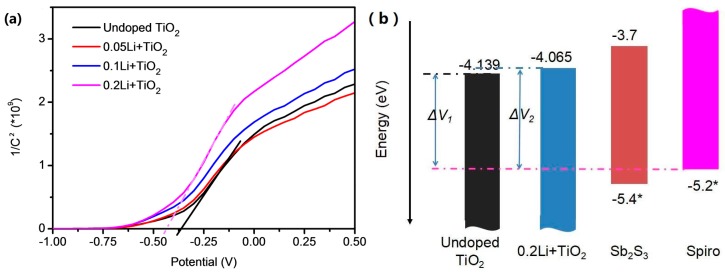
(**a**) Mott–Schottky curves of the mesoporous TiO_2_ varying with Li-doping. (**b**) Energy band scheme [32].

Because Fermi level is one of the most important semiconductor properties of TiO_2_, e.g., the difference of Fermi level in value between TiO_2_ and the highest occupied molecular orbital level of the hole, transport layers can determine the open circuit voltage and charge extraction of solar cells [31]. We primarily characterized the Fermi level of the TiO_2_ films to further evaluate the influence of Li-doping on the electron dynamics in the mesoporous TiO_2_ layers. As shown in Figure 6a, the Mott–Schottky curves are fitted by the following equation:

(1) $$\frac{1}{C^{2}}=\frac{2}{e\varepsilon \varepsilon_{0}N_{D}}(E-E_{f}-\frac{kT}{e})$$

where *C* represents the capacitance of the space charge region; *E* is applied potential, *E_f_* is the Fermi level potential, and *e*, *ε*, *ε*_0_, *k*, *T* represent the electron charge, the dielectric constants of materials, the vacuum permittivity, the Boltzmann constant and the absolute temperature, respectively. As shown in Figure 6a, when the potential is applied, the capacitance of the lithium-doped TiO_2_ films changes faster than that of the undoped TiO_2_, suggesting that a faster charge transport can happen from absorbing layer to electron transport layer in the solar cells. Besides the enhanced charge transport, the Fermi level must be another important reason for the improved photovoltaic performance. The fitted flat band potential for the Li-doped TiO_2_ is −0.435 eV, for the undoped TiO_2_ it is −0.361 eV. Correspondingly, an energy band diagram is presented in Figure 6b, where the difference of potentials between 0.2Li + TiO_2_ and Spiro (*ΔV*_2_) is higher than that between undoped TiO_2_ and Spiro (*ΔV*_1_). Therefore to some extent, the difference of energy bands can explain the improved *V_oc_* of the Sb_2_S_3_ solar cells based on the Li-doped mesoporous TiO_2_ layers.

## 4. Conclusions

In conclusion, we studied the effect of lithium-doping on charge transport properties of mesoporous TiO_2_ layers for thermal-evaporated Sb_2_S_3_ thin film solar cells. XPS results demonstrated that lithium has been successfully introduced into the TiO_2_. Based on the Mott-Schottky curves of the mesoporous TiO_2_, it was found that lithium doping raised their platform potential and enhanced the charge transport. With the lithium doping on mesoporous TiO_2_, the photovoltaic performance of the thermal-evaporated Sb_2_S_3_ solar cells has been dramatically improved. Compared with the solar cells using undoped TiO_2_, the solar cells with Li-doped TiO_2_ apparently demonstrated higher average *PCE*, from 1.79% to 4.04%, as well as the champion *V_oc_* from 0.595 to 0.645 V, *J_sc_* from 13.8 to 15.04 mA/cm^2^ and the increased shut resistance. The Sb_2_S_3_ solar cells based on 0.2 M lithium-doped TiO_2_ reached the champion *PCE* of 4.42%, which is the highest *PCE* among the reported thermal-evaporated Sb_2_S_3_ solar cells, and they showed less variation. This study provided a new strategy to improve the photovoltaic performance of Sb_2_S_3_-based solar cells.

## Figures and Tables

**Table 1:** Photovoltaic parameters of the thermal-evaporated Sb_2_S_3_ solar cells based on different mesoporous TiO_2_, measured under one Sun AM 1.5G illumination.

Mesoporous TiO_2_		*V_oc_* (V)	*J_sc_* (mA/cm^2^)	FF	*PCE* (%)	*R_s_* (Ω cm^2^)	*R_sh_* (Ω·cm^2^)
Undoped-TiO_2_	champion	0.595	13.8	0.45	3.74	87	168
	Average	0.591	10.4	0.28	1.79	-	-
0.05Li-TiO_2_	champion	0.595	13.2	0.41	3.19	57	100
	Average	0.606	10.9	0.30	1.93	-	-
0.1Li-TiO_2_	champion	0.635	14.1	0.45	4.03	69	123
	Average	0.606	13.5	0.45	3.74	-	-
0.2Li-TiO_2_	champion	0.635	15.0	0.46	4.42	68	149
	Average	0.629	14.3	0.45	4.03	-	-

## Supplementary Materials

The following are available online at http://www.mdpi.com/1996-1944/11/3/355/s1.
