# Supplementary material for: Enhanced Charge Extraction of Li-Doped TiO2 for Efficient Thermal-Evaporated Sb2S3 Thin Film Solar Cells
Source: Materials (Basel). 2018 Feb 28;11(3):355. doi: 10.3390/ma11030355 (PMC5872934; doi:10.3390/ma11030355)
Supplement: Supplementary file 1 [file materials-11-00355-s001.pdf]

materials

Supplementary

Enhanced Charge Extraction of Li-Doped TiO_2 for Efficient Thermal-Evaporated Sb_2S_3thin Film Solar Cells

Chunfeng Lan [1,2,3,†], Jingting Luo [1,3,†], Huabin Lan [1,3], Bo Fan [1,3], Huanxin Peng [1,3], Jun Zhao [1,3], Huibin Sun [1,2], Zhuanghao Zheng [1,3], Guangxing Liang [1,3,*] and Ping Fan [1,3,*]

[1] Shenzhen Key Laboratory of Advanced Thin Films and Applications, College of Physics and Energy, Shenzhen University, Shenzhen 518060, China; lanchunfeng@gmail.com (C.L.); luojt@szu.edu.cn (J.L.); lanhb420@163.com (H.L.); fanb07@hotmail.com (B.F.); P2385284535@163.com (H.P.); zhaojun@szu.edu.cn (J.Z.); hbsun@szu.edu.cn (H.S.); zhengzh@szu.edu.cn (Z.Z.)

[2] Key Laboratory of Optoelectronic Devices and Systems of Ministry of Education and Guangdong Province, College of Optoelectronic Engineering, Shenzhen University, Shenzhen 518060, China

[3] Institute of Thin Film Physics and Applications, College of Physics and Energy, Shenzhen University, Shenzhen 518060, China

† These authors contributed equally to this work.

* Corresponding author: lgx@szu.edu.cn (G.L.); fanping@szu.edu.cn (P.F.)

Received: 16 January 2018; Accepted: 26 February 2018; Published: 28 February 2018

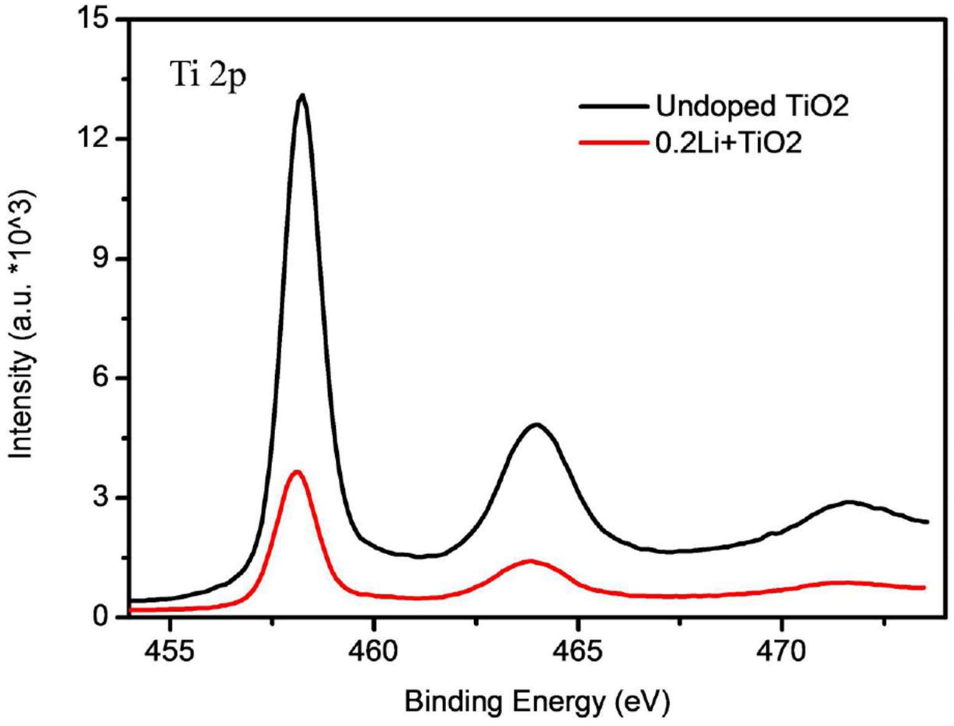

Figure S1. XPS results of TiO_2 on Ti2p.

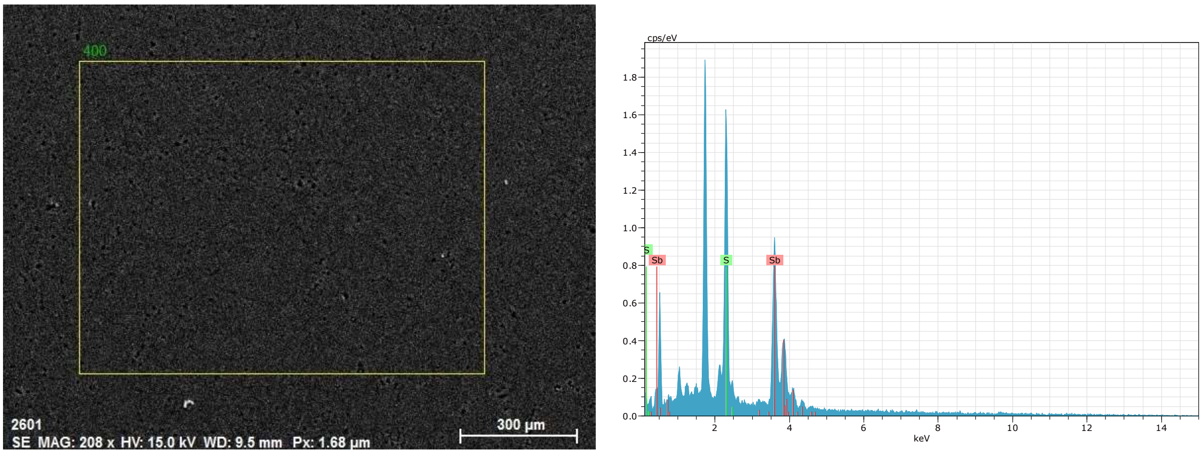

Figure S2. EDX mapping of the annealed thermal-evaporated Sb_2S_3 films.

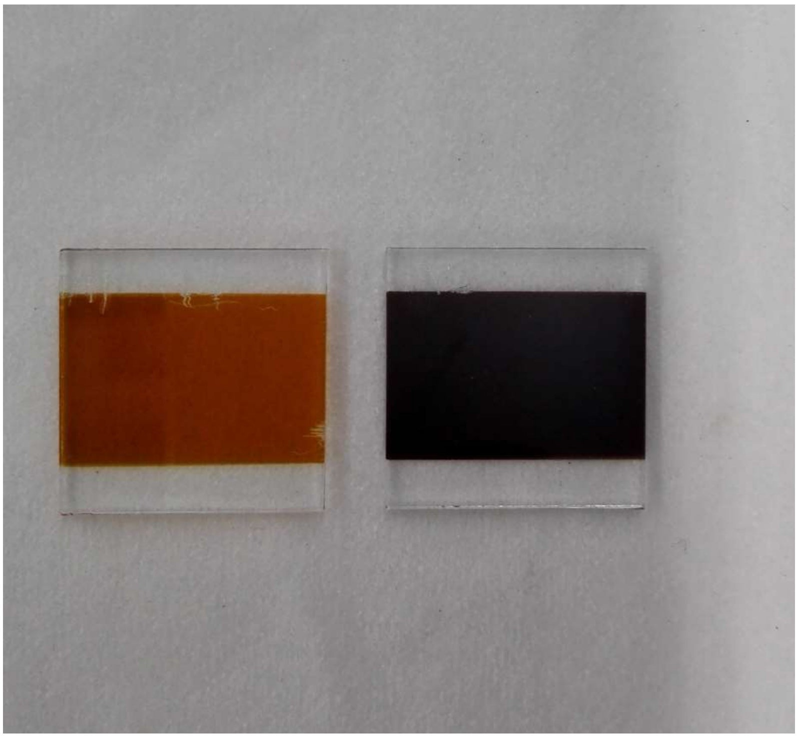

Figure S3. Samples of the thermal-evaporated Sb_2S_3 films: left is the as-deposited film, right is the annealed film.

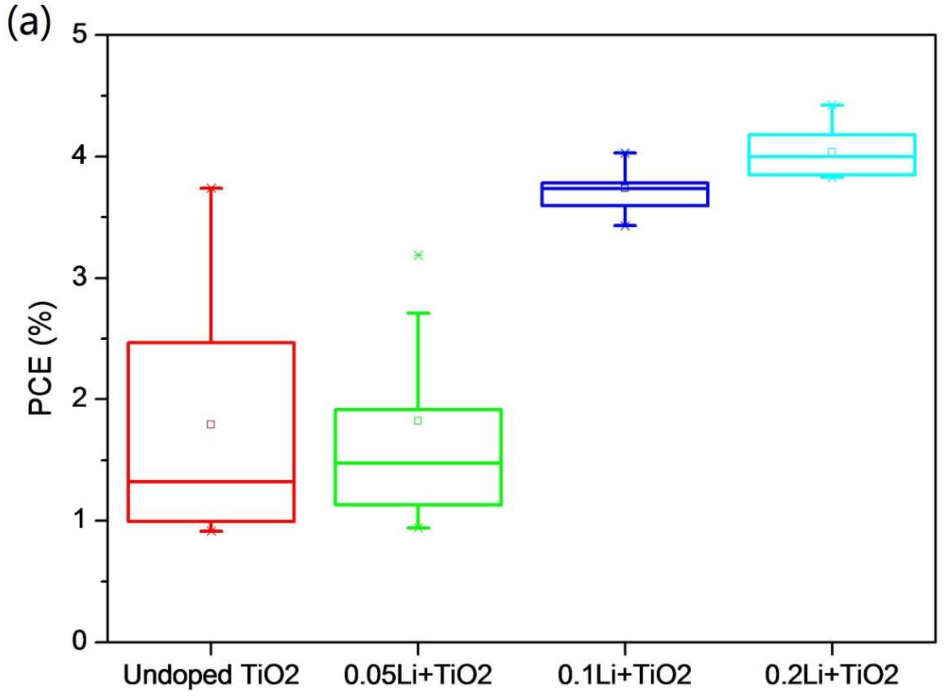
(a)
PCE (%)
5
4
3
2
1
0
Undoped TiO2
0.05Li+TiO2
0.1Li+TiO2
0.2Li+TiO2

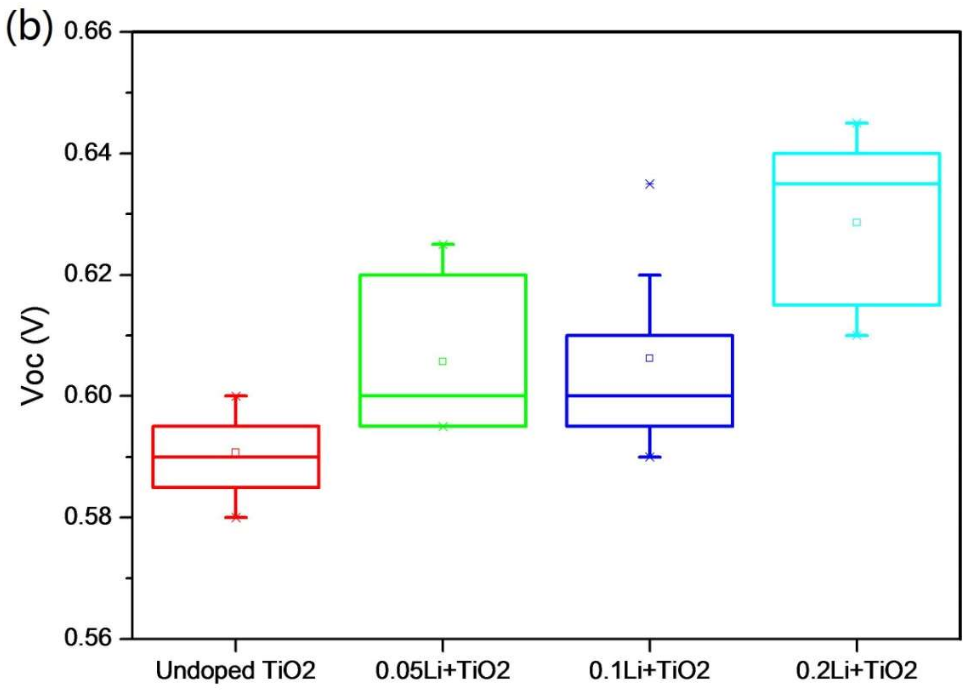
(b)
Voc (V)
0.66
0.64
0.62
0.60
0.58
0.56
Undoped TiO2
0.05Li+TiO2
0.1Li+TiO2
0.2Li+TiO2

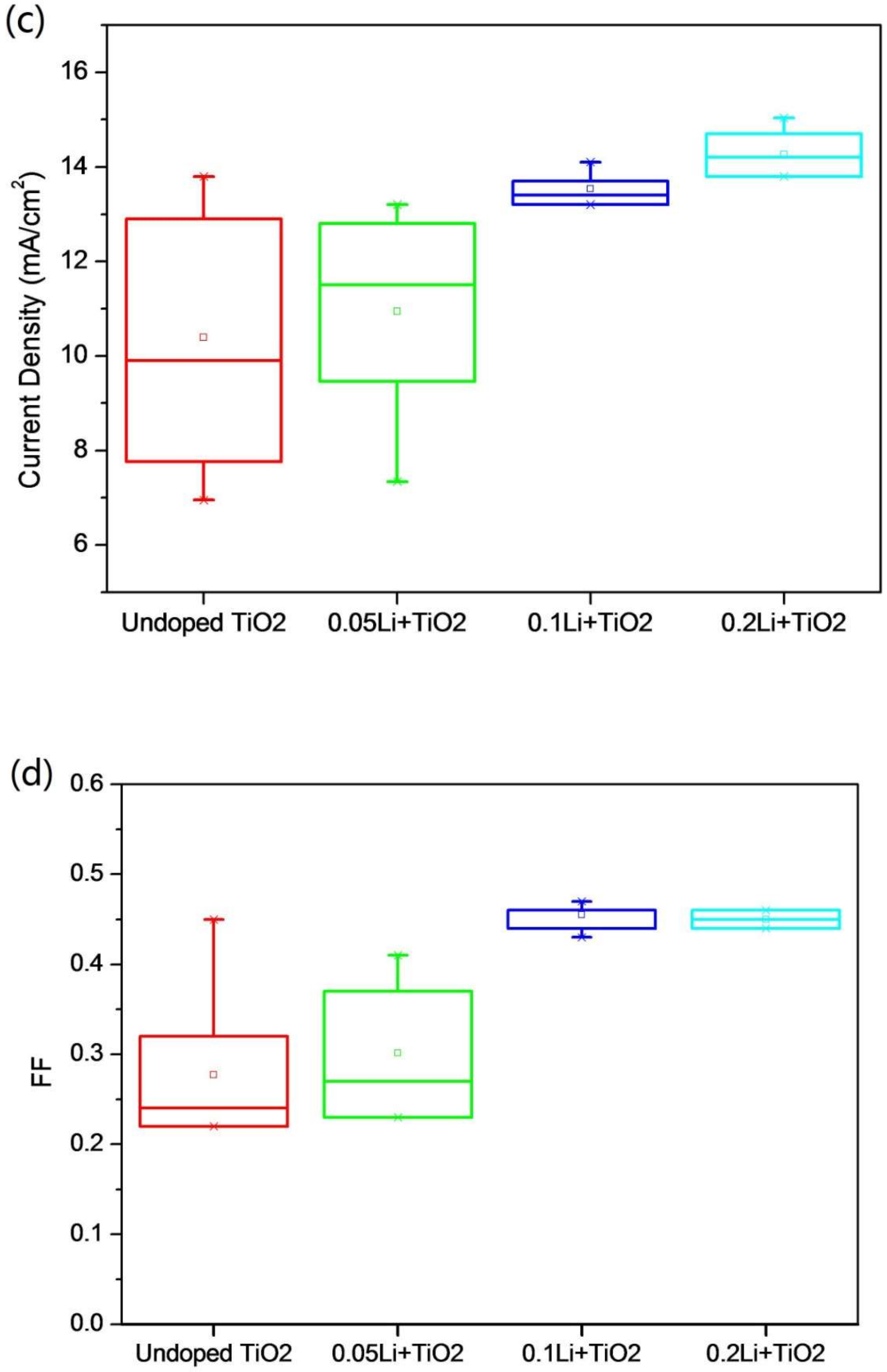

Figure S4. Statistics of *PCE* (a), V_{oc} (b), J_{sc} (c) and *FF* (d) of the devices.

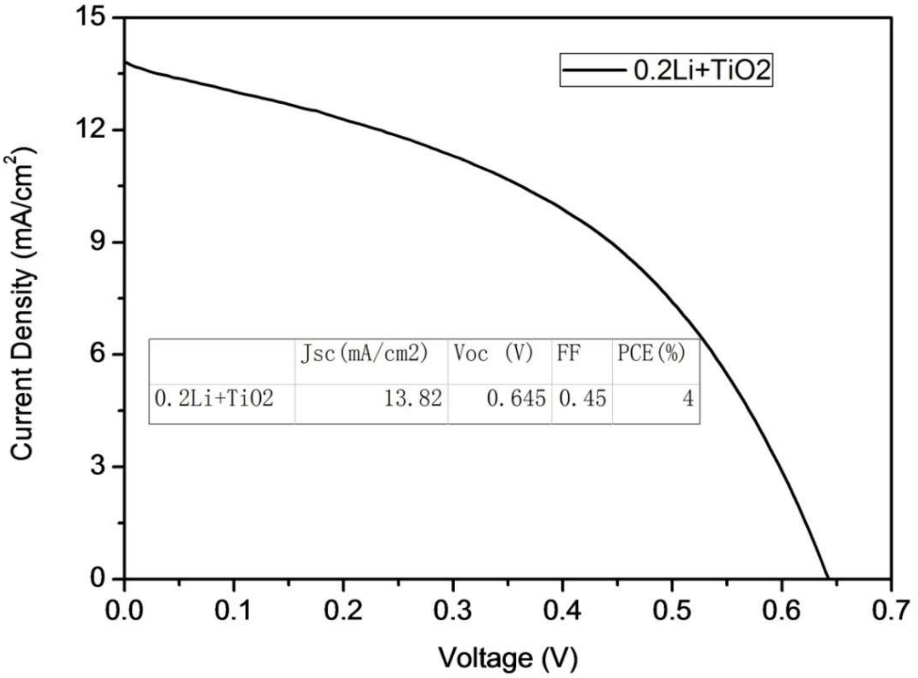

Figure S5. The *J-V* curve of the devices based on 0.2Li + TiO_2 with highest V_{oc}.

Table S1. Photovoltaic parameters of thermal-evaporated Sb_2S_3 solar cells with different Li-doped TiO_2.

**Mesoporous TiO_2**	R_s **(Ω·cm²)**	R_{sh} **(Ω·cm²)**	*PCE* **(%)**	*FF*	V_{oc} **(V)**	J_{sc} **(mA/cm²)**
undoped TiO_2						
	14.7	41	1.88	0.27	0.585	11.82
	20	40	1.23	0.22	0.580	9.62
	36	52	0.99	0.22	0.595	7.76
	19	51	2.46	0.32	0.590	12.9
	19	41	1.32	0.24	0.590	9.9
	34	75	0.91	0.22	0.600	6.95
	87	168	3.74	0.45	0.595	13.8
Average	32.8	66.9	1.79	0.28	0.591	10.39
0.05Li-TiO_2						
	14	39	1.37	0.23	0.595	10.59
	16	87	1.13	0.25	0.620	7.34
	9	48	1.36	0.23	0.625	9.46
	15	43	1.84	0.37	0.605	11.5
	22	64	2.7	0.35	0.600	12.8
	57	100	3.19	0.41	0.595	13.2
	18	42	1.91	0.27	0.600	11.7
Average	21.6	60.4	1.93	0.30	0.606	10.94
0.1Li-TiO_2						

	91	139	3.43	0.44	0.590	13.2
	81	154	3.59	0.46	0.595	13.2
	103	166	3.73	0.46	0.600	13.4
	76	117	3.73	0.43	0.620	14.0
	84	167	3.75	0.47	0.600	13.3
	102	165	3.78	0.47	0.600	13.4
	71	141	3.86	0.46	0.610	13.7
	69	123	4.03	0.45	0.635	14.1
Average	84.6	146.5	3.74	0.455	0.606	13.54
0.2Li-TiO_2						
	68	149	4.42	0.46	0.635	15.0
	65	134	4.00	0.45	0.645	13.8
	50	123	3.83	0.44	0.620	13.9
	78	143	3.94	0.45	0.615	14.2
	77	130	4.02	0.45	0.610	14.7
	49	117	3.85	0.44	0.640	13.8
	82	142	4.18	0.46	0.635	14.4
Average	67.0	134	4.03	0.45	0.629	14.26
